# Factors associated with graft survival and endothelial cell density after Descemet’s stripping automated endothelial keratoplasty

**DOI:** 10.1038/srep25276

**Published:** 2016-04-28

**Authors:** Nobuhito Ishii, Takefumi Yamaguchi, Hiroyuki Yazu, Yoshiyuki Satake, Akitoshi Yoshida, Jun Shimazaki

**Affiliations:** 1 Department of Ophthalmology, Ichikawa General Hospital, Tokyo Dental College, Chiba, Japan; 2 Department of Ophthalmology, Asahikawa Medical University, Asahikawa, Japan

## Abstract

Postoperative endothelial cell loss leads to graft failure after corneal transplantation, and is one of the important issues for long-term prognosis. The objective of this study was to identify clinical factors affecting graft survival and postoperative endothelial cell density (ECD) after Descemet’s stripping automated endothelial keratoplasty (DSAEK). A total of 198 consecutive Japanese patients (225 eyes) who underwent DSAEK were analysed using Cox proportional hazard regression and multiple linear regression models. The candidate factors included recipient age; gender; diagnosis; pre-existing iris damage state, scored based on its severity; the number of previous intraocular surgeries; graft ECD; graft diameter; simultaneous cataract surgery; surgeons experience; intraoperative iris damage; postoperative rebubbling; and graft rejection. Eyes with higher pre-existing iris damage score and more number of previous intraocular surgery had a significantly higher risk of graft failure (HR = 8.53; P < 0.0001, and HR = 2.66; P = 0.026, respectively). Higher pre-existing iris damage score, lower graft ECD, and smaller graft diameter were identified as significant predisposing factors for lower postoperative ECD. The results show that iris damage status before DSAEK may be clinically useful in predicting the postoperative course. Avoiding intraoperative iris damage, especially in eyes with low ECD can change the prognosis of future DSAEK.

Descemet’s stripping automated endothelial keratoplasty (DSAEK) for the treatment of endothelial dysfunction has several advantages over standard penetrating keratoplasty (PKP)^1^,^2^,^3^. By removing only Descemet’s membrane and dysfunctional endothelium, and retaining healthy portions of the patient’s cornea, DSAEK offers more rapid visual recovery and preservation of the cornea’s biomechanical properties and integrity^4^,^5^. In addition, it provides excellent vision in most patients by reducing higher-order aberrations and improving corneal transparency^6^,^7^,^8^,^9^, resulting in less graft rejections than PKP^9^,^10^,^11^,^12^, and leading to a favourable long-term graft survival rate up to 85–87% at 5 years^10^,^11^. However, endothelial cell density (ECD) decreases after DSAEK as in eyes after PKP, which leads to endothelial dysfunction even without graft rejection^13^,^14^. Thus, to prolong graft survival and maintain good long-term vision after DSAEK, postoperative endothelial cell loss is an important issue that has to be addressed.

ECD decreases over time after corneal transplantation^15^,^16^,^17^,^18^, and risk factors for postoperative endothelial cell loss after PKP include donor age, recipient age, graft diameter, lens status, the presence of glaucoma, graft rejection, and peripheral corneal diseases^18^,^19^,^20^,^21^. However, the factors influencing endothelial cell loss after DSAEK are still poorly understood. ECD rapidly decreases in some patients after DSAEK, with no apparent intra- or postoperative complications. Recently, we noticed a rapid postoperative ECD decrease in some patients with severe pre-existing iris damage, whereas the ECD decrease was minimal in patients with a healthy iris. Thus, we hypothesized that iris damage can lead to rapid endothelial cell loss after DSAEK. In this study, using Cox proportional hazard regression and multiple linear regression, we evaluated potential factors that might be related to the risk of corneal graft failure and postoperative lower ECD, focusing especially on the pre-existing iris damage status.

## Results

### Patient Demographics

**Figure 1:**
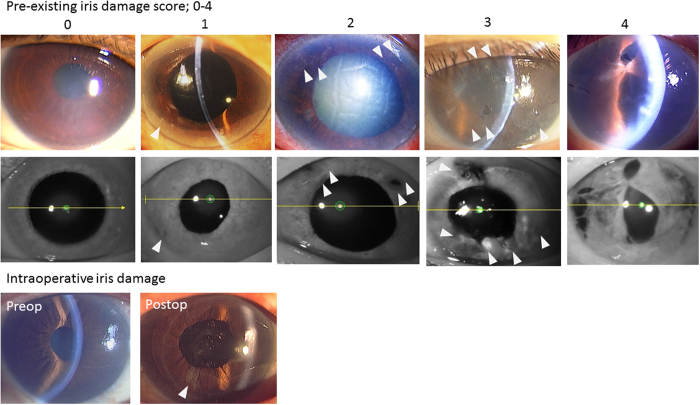
Iris damage scoring methods. Iris damage (white arrows) was scored based on preoperative slit-lamp microscopy and iris photograph using infrared light as follows: 0, no iris damage; 1, iris damage limited to only one quadrant: 2, iris damage in two quadrants: 3, iris damage in three quadrants: 4, iris damage in four quadrants. Intraoperative iris damage was assessed and regarded as one of independent factors.

Table 1 shows the demographics of the recipients, the donors of DSAEK graft, and intra- and postoperative characteristics. The mean age of the recipients was 69.7 ± 12.1 years old. Pre-existing iris damage score was determined based on its severity from preoperative slit-lamp microscopy findings as follows: 0, no iris damage; 1, iris damage limited to only one quadrant; 2, iris damage in two quadrants; 3, iris damage in three quadrants; and 4, iris damage in four quadrants (Fig. 1). Pre-existing iris damage scores were 0 in 104 eyes, 1–2 in 89 eyes, and 3–4 in 31 eyes. Graft diameter were determined by surgeons, based on the patients’ corneal diameter, and 8.0 mm diameter was most common diameter in the current study. Solitary DSAEK was performed in 143 eyes and simultaneous DSAEK and cataract surgery was performed in 82 eyes. Intraoperative iris damage occurred in 27 eyes of 27 patients, which was regarded as an independent factor from the pre-existing iris damage. Postoperative rebubbling was performed to treat postoperative double chamber, graft dislocation or graft detachment.

### Visual Outcomes and ECD

Logarithm of minimal angle resolution (logMAR) visual acuity improved significantly, from 1.18 ± 0.57 to 0.48 ± 0.54 at 3 months, 0.34 ± 0.35 at 6 months, 0.31 ± 0.35 at 12 months, and 0.26 ± 0.32 at 24 months (all P < 0.0001, one-way ANOVA, Tukey’s multiple comparisons post-test). The average graft ECD decreased significantly, from 2651 ± 323 cells/mm^2^ to 1332 ± 550 at 3 months, 1244 ± 520 at 6 months, 1104 ± 545 at 12 months, 949 ± 499 at 24 months, and 822 ± 531 at 36 months (all P < 0.0001, one-way ANOVA, Tukey’s multiple comparisons post-test). The ECDs at 12, 24, and 36 months were significantly lower than that at 3 months (P < 0.01). The ECD at 36 months was significantly lower than those at 6 and 12 months (P < 0.001).

### Predictors of graft failure in all patients

Table 2 shows the results of Cox proportional hazard regression. Univariate models showed that pre-existing iris damage scores of 3 or 4, and the number of previous intraocular surgeries were significantly associated with graft failure (HR, 8.53; 95% CI, 3.60–22.4; P < 0.0001, and HR, 2.66; 95% CI, 1.25–5.31; P = 0.026, respectively). The multifactorial analyses showed that the following factors were significantly associated with graft failure; pre-existing iris damage scores (HR, 7.57; 95% CI, 2.57–24.3; P = 0.0002), inexperienced surgeon (HR, 2.51; 95% CI, 1.03–6.09; P = 0.042), transscleral suturing of the intraocular lens (TS-IOL) (HR, 3.88; 95% CI, 1.03–12.4; P = 0.046), and postoperative rebubbling (HR, 2.71; 95% CI, 1.06–6.32; P = 0.037). Multiple linear regression analyses showed that pre-existing iris damage scores, preoperative graft ECD and graft diameter were predisposing factors that had significant correlations with postoperative ECD at all time points (Table 3).

### Predictors of graft failure in uncomplicated patients

We postulated that pre-existing iris damage may be associated with a history of trabeculectomy and PKP. Furthermore, potential influential factors, such as the presence of diabetes mellitus (DM), graft rejection, postoperative rebubbling and uveitis could affect the above results and cause confounding bias. To completely exclude the influence of these factors on graft failure and postoperative endothelial cell density (ECD), the subgroup was defined as the patients who underwent DSAEK due to laser-iridotomy-related bullous keratopathy (LI-BK), pseudophakic/aphakic bullous keratopathy (PBK/ABK), Fuchs’ endothelial corneal dystrophy (FECD), the three major indications for DSAEK, excluding the patients with DM, intraoperative iris damage, uveitis, postoperative rebubbling, and rejection. Supplementary Table S1 shows the demographics of the subgroup. Univariate and multifactorial analysis showed that pre-existing iris damage scores of 3 or 4 were significantly associated with graft failure (Supplementary Table S2, HR, 13.6; 95% CI, 1.54–119; P = 0.019). Stepwise multiple linear regression analysis showed that the predisposing factors that had significant correlations with postoperative ECD were pre-existing iris damage scores at 3, 6, and 12 months, the recipients’ age at surgery at 3 and 24 months, graft ECD at 6, 12, and 24 months, and the graft diameter at 6 and 12 months (Supplementary Table S3).

### Influence of Iris Damage Score on Graft Survival

**Figure 2:**
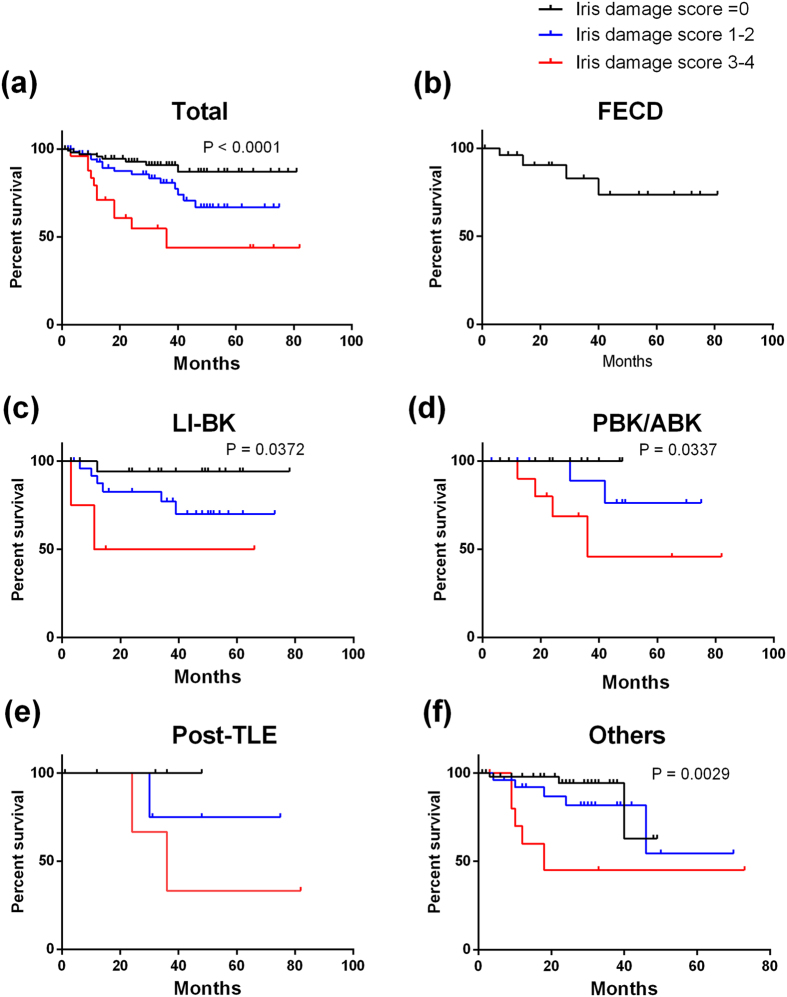
Kaplan Meier survival curves stratified by iris damage score and different aetiologies. In all subjects (**a**) and the subjects with LI-BK (**c**) PBK (**d**) and other aetiologies (**e**) there were significant differences in survival rates among the subjects with no iris damage or low iris damage score (1–2) and those with high iris damage scores (3–4). In eyes with FECD, no patient had iris damage. Others included failed graft after PKP (12 eyes) and failed DSAEK (10 eyes), chronic uveitis (6 eyes), birth injury (5 eyes), and other causes such as endotheliitis.

Because the iris damage score was the most potent influential factor for postoperative endothelial cell loss, we stratified the subjects based on iris damage scores in different aetiologies. In total subjects (a), eyes with LI-BK (c), PBK (d), and other aetiologies (f), the survival rates in eyes with iris damage scores of 3 or 4 were significantly worse than those of eyes with either no iris damage or iris damage scores of 1–2 (Fig. 2; P < 0.0001, P = 0.037, P = 0.0337, and P = 0.0029, respectively).

### Influence of Iris Damage Score on ECD Loss

**Figure 3:**
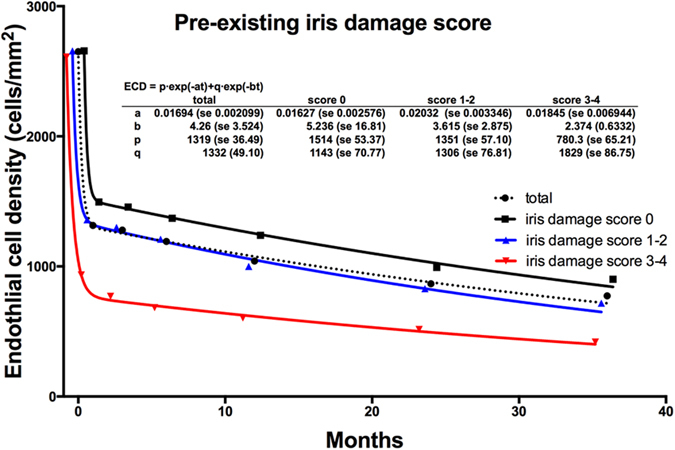
Endothelial cell loss after DSAEK classified by iris damage score. A biexponential model was fitted to the ECD classified by the iris damage score. The decrease in the ECD was greater in the group with iris damage scores of 3–4 than the groups with iris damage score of 0 and 1–2.

To evaluate the effect of iris damage scores on the time course decrease in postoperative ECD after DSAEK, we used an exponential model^16^, classifying the subjects based on the iris damage score (Fig. 3). The eyes with iris damage score of 3–4 showed a rapid decrease in ECD, compared with the groups with iris damage score of 0 and 1–2.

## Discussion

The results demonstrated that graft failure was associated with pre-existing iris damage score, and that ECD after DSAEK was correlated with preoperative graft ECD, graft diameter, and pre-existing iris damage of recipients, suggesting that endothelial cell loss after DSAEK is influenced by both donor and recipient factors.

Although the overall clinical outcomes including visual acuity, astigmatism, and ammetropia after DSAEK are favourable compared to PKP, the survival rate has been reported to be 80–95% and endothelial cell loss is the major cause of graft failure. Many sophisticated surgical techniques have been applied to prevent endothelial cell loss during surgery and have contributed to the better clinical outcomes^22^,^23^,^24^. However, we experienced rapid endothelial cell loss in a small proportion of patients after DSAEK, even when the procedure was performed in the same way with no intra- and postoperative complications. Factors related to endothelial cell loss after PKP have been well documented^18^,^19^,^20^,^21^. However, to the best of our knowledge, the clinical factors for endothelial cell loss after DSAEK is poorly understood. We hypothesized that some pre-disposing factors must exist in donors, surgical methods, postoperative complications, or the recipients’ eyes.

Under normal conditions, the adult human cornea loses endothelial cells at a rate of 0.6% per year^25^. After cataract surgery, the annual rate of endothelial cell loss is 2.5% per year between 1 and 10 years after surgery^26^. After PKP with no complications, endothelial cells decrease at a rate of 2.6–7.8% per year^27^,^28^,^29^, and both donor and recipient factors associated with postoperative ECD have been identified in eyes after PKP. Donor factors, younger age, larger preoperative graft ECD, larger donor graft diameter, and female donors are factors that correlate with larger postoperative ECD^15^,^17^,^19^. As recipient factors for endothelial cell loss after PKP, several studies have reported that eyes with anterior chamber IOL (AC-IOL), glaucoma, or ocular hypertension have lower ECD and higher failure rates^18^,^21^,^27^,^30^,^31^,^32^.

In the current study, we identified pre-existing iris damage as a recipient factor associated with less ECD after DSAEK. Moreover, the higher the pre-existing iris damage score, the higher the incidence of graft failure. Although the association between iris damage and ECD is a novel result, some clinical entities with iris pigment atrophy, such as iridocorneal endothelial syndrome, have progressive endothelial cell loss^33^,^34^. The presence of pre- and intraoperative iris damage can cause prolonged postoperative intraocular inflammation, due to the breakdown of the blood-aqueous barrier, which may lead to endothelial cell loss, because previous studies have implied a correlation between ocular inflammation and endothelial cell loss^35^. Severe anterior segment inflammation is related to iris atrophy^36^. Cytokines, interleukin-1, tumor necrosis factor, and vascular endothelial growth factor are elevated in the aqueous humor of the eyes with bullous keratopathy (BK)^37^ and in eyes after trabeculectomy^38^, which is known as one of the risk factors for graft failure after DSAEK. Severe corneal or anterior chamber inflammation can result in concomitant endothelial cell loss and BK^41^. It is noteworthy that pre-existing iris damage was associated with decreased ECD and higher graft failure, which indicates that patients with pre-existing iris damage should be advised about higher risk for graft failure before DSAEK. Specific mechanisms have to be substantiated by comprehensive studies, to determine the sequence of events that take place in eyes with iris damage during and after DSAEK with regard to the apoptotic factors in the aqueous humor and inflammatory reactions in endothelial cells. It is tempting to speculate that the iris epithelium may secrete some factors that maintain endothelial cell survival.

Other factors, such as the number of previous surgeries and the history of TS-IOL and LI should be evaluated as the factors for ECD loss after DSAEK. The number of previous intraocular surgeries was associated with a higher incidence of graft failure in the current study, not with postoperative ECD. Furthermore, there was a significant correlation between iris damage scores and the number of previous intraocular surgeries (R = 0.262, P < 0.001). Therefore, iris damage during previous intraocular surgery can worsen the prognosis of future DSAEK, suggesting that iris damage should be avoided during intraocular surgery, especially in eyes with low ECD. The lens status is a well-known influential factor for ECD after PKP; the prognosis is poor in eyes with AC-IOL^18^,^19^,^20^,^21^. In the current study, because we performed AC-IOL removal with TS-IOL in all eyes with AC-IOL bullous keratopathy prior to DSAEK, we did not include DSAEK cases with AC-IOL. We previously evaluated the outcome of DSAEK in eyes with TS-IOL, comparing with solitary DSAEK^42^. Although there was no statistical difference in the ECDs between the groups up to 24 months after DSAEK, we found rapid decreases in postoperative ECD in several eyes with severe iris damage in the DSAEK group after TS-IOL. Regarding the presence of LI, we previously reported that the prognosis of DSAEK in eyes with LI is comparable with that of FECD and PBK^43^. In the current study, the presence of LI was not associated with the graft survival and postoperative ECD. Although the prognosis of DSAEK in eyes after some types of glaucoma surgeries, such as trabeculectomy, is not favourable, the results of this study suggest that the presence of LI does not affect graft survival and endothelial cell loss.

We demonstrated that larger graft diameter and more preoperative graft ECD were associated with more ECD after DSAEK. Romano *et al.*^44^ reported that larger graft diameter and higher donor ECD were significantly associated with a reduced graft failure rate. Infant donors with a preoperative graft ECD of more than 4000 cell/mm^2^ were found to be preferable for higher ECD after DSAEK^45^,^46^. The results of the current study are consistent with these reports, although there is still a controversy on the association between ECD and graft diameter^47^. It is natural that a larger graft diameter is associated with higher ECD after DSAEK, because ECD is higher in the peripheral cornea than at the center of the cornea, and because a graft diameter of 8.5 mm is larger, by 28%, than a graft diameter of 7.5 mm, and is therefore theoretically expected to provide more endothelial cells. However, in eyes after PKP, a larger graft leads to a higher incidence of graft rejection. The more antigens that are transplanted, the higher the incidence of graft rejection, as has been reported in the comparison of graft rejection among PKP, DSAEK and Descemet’s membrane endothelial keratoplasty^48^. In the future, studies on the incidence of graft rejection in eyes with different graft diameters will be important in confirming the safety of larger DSAEK grafts.

Our study had several limitations. First, we excluded the history of trabeculectomy (9 eyes, 3.9%) from the baseline factors of multifactorial analysis because there were too few cases after trabeculectomy. Post-trabeculectomy has been reported to be associated with graft failure after DSAEK^49^,^50^. The univariate analysis using the Cox proportional analysis showed that the hazard ratio of post-trabeculectomy for graft failure was 3.23 (95% CI = 1.14–9.09; P = 0.027), which is consistent with previous studies. Furthermore, Inoue *et al.* reported that cytokine levels, such as monocyte chemotactic protein -1, in the aqueous humor were elevated in eyes with trabeculectomy^38^. Future studies have to be conducted to elucidate the associations between the cytokine levels in the aqueous humor, iris damage score and postoperative endothelial cell loss after DSAEK. Second, the decreased ECD can be influenced by the experience of the surgeon. In the current study, the involvement of an experienced surgeon was correlated with graft survival, but not with postoperative ECD.

In conclusion, the results of the current study suggest that a larger graft diameter is important in maintaining higher postoperative ECD, thereby providing more endothelial cells. Postoperative endothelial cell loss and graft failure were associated with higher pre-existing iris damage scores as recipient factors. Pre-existing iris damage scores can be useful in predicting the prognosis of DSAEK in patients with BK.

## Methods

This retrospective cohort study was conducted in accordance with the Declaration of Helsinki, and was approved by the Ethics Review Board of Tokyo Dental College Ichikawa General Hospital (Acceptance No. I-15-39). Informed consent was obtained from all the subjects.

### Subjects

Consecutive patients who underwent DSAEK between March 2007 and December 2011 at the Department of Ophthalmology, Ichikawa General Hospital, Tokyo Dental College, were studied. A total of 225 eyes from 198 patients (66 males and 132 females) were enrolled (Table 1). The causes of BK included LI (81 eyes, 36%), PBK/ABK (54 eyes, 24%), FECD (26 eyes, 12%), after trabeculectomy (10 eyes, 4%), failed graft after PKP (12 eyes, 5%) and failed DSAEK (10 eyes, 4%), birth injury (5 eyes, 2%), and other causes such as endotheliitis and chronic uveitis.

### Surgical technique

DSAEK surgery was performed using double-glide technique^51^. After retrobulbar anesthesia with injection of 2% lidocaine, a 5.0-mm temporal corneoscleral incision was made. An AC maintenance cannula was inserted through the 2 or 10 o’clock paracentesis, and Descemet stripping was performed with a reverse-bent Sinsky hook (Asico, Westmont, IL, USA). The recipient’s endothelium and Descemet’s membrane were carefully removed using forceps. Pre-cut donor grafts were trephinated at a diameter of 7.0–8.5 mm, and the endothelial surface of the donor lenticle was coated with a small amount of viscoelastic material. Donor tissue was gently inserted into the anterior chamber using a Busin glide (Asico) and Shimazaki DSAEK forceps (Inami, Tokyo, Japan). Air was carefully injected into the anterior chamber to unfold the graft. The fluid between the recipient’s stroma and the graft was drained from small incisions in the midperipheral recipient cornea. At 10 min after air injection, half of the air was replaced by balanced salt solution (BSS, Alcon, Fort Worth, TX, USA). At the end of the surgery, 2 mg subconjunctival betamethasone was administered. In patients with significant lens opacity (82 eyes), standard phacoemulsification and aspiration were performed using the phaco-chop technique with implantation of an IOL, followed by the DSAEK procedure. Postoperative medications included levofloxacin (Cravit, Santen, Osaka, Japan) and 0.1% betamethasone sodium phosphate (Sanbetazon, Santen), which were prescribed five times per day for 3–6 months. Topical 0.1% fluorometholone was prescribed two times per day after cessation of topical betamethasone.

### Data Analysis

Outcome measures included best-corrected visual acuity (BCVA), postoperative ECD and graft survival rate. The standard Landolt optotype chart was used to measure BCVA, which was converted into the logMAR and analyzed statistically. ECD was measured preoperatively and at 1, 3, 6, 12, and 24 months after surgery using a non-contact specular microscope (Noncon Robo SP-8000, Konan, Hyogo, Japan). Approximately 50 cells were analyzed for mean cell density. Graft failure was defined as persistent corneal edema resulting in irreversible loss of optical clarity due to the decrease in ECD.

To identify predictive parameters that are associated with postoperative ECD and graft failure, we selected the following variables, that were a priori based on the past studies and our knowledge of ECD and DSAEK; recipient gender, age, history of DM, aetiologies of BK, the number of previous intraocular surgeries, IOL position [capsular bag or TS-IOL] and pre-existing iris damage score were selected as the recipient factors. Donor age, imported or domestic graft, graft ECD and graft diameter were selected as the donor factors. Simultaneous cataract surgery, the surgeon’s experience, intraoperative iris damage, postoperative rebubbling and rejection episodes were selected and analyzed as the intra- and postoperative factors.

Two masked observers (NI and TY) determined the iris damage score, based on its severity from preoperative slit-lamp microscopy findings (Fig. 1) as follows: 0, no iris damage; 1, iris damage limited to only one quadrant; 2, iris damage in two quadrants; 3, iris damage in three quadrants; and 4, iris damage in four quadrants. In Asian eyes with brown pigmentation, the iris damage was easily identified as the depigmented area using the slit-lamp microscopy. In all eyes, pre-existing iris damage scores based on the preoperative slit-lamp microscopy were confirmed using infrared camera photograph of swept source anterior segment optical coherence tomography (AS-OCT, CASIA, SS-1000; Tomey, Nagoya, Japan), because it was difficult to score precisely in some eyes due to the severe corneal edema before DSAEK. If the iris damage score increased after DSAEK, we regarded it as intraoperative iris damage, which was one of the other independent candidate factors for graft failure and postoperative ECD. LI was regarded as a factor independent from iris damage score, because not all patients who underwent LI developed BK and because we had sufficient numbers of patients who underwent LI (81 eyes); we were able to analyse them as an independent factor for Cox proportional hazard regression analyses.

An experienced surgeon was defined as a surgeon who performed more than 30 DSAEK procedures. Factors, such as gender, presence of DM, graft rejection, an experienced surgeon, and postoperative rebubbling were dichotomized for Cox proportional hazards regression. Continuous variables including recipient age, donor age, and graft ECD were also dichotomized for use as categorical variables for Cox proportional hazards regression.

### Statistical analysis

Data were analyzed using the SPSS software (ver. 23; SPSS, Inc, Chicago, IL). To identify predisposing factors associated with graft failure, Cox proportional hazards regression was first used (univariate analysis and multifactorial analysis). Univariate analysis was performed to determine the correlation between graft failure and each variable. In multifactorial analysis, all of the variables were included to evaluate the relationship with graft failure. To exclude the influence of factors such as post-trabeculectomy and other ocular complicated pathologies, we focused on three major indications for DSAEK in Japan; LI-BK, PBK and FECD. Eyes with graft rejection, intraoperative iris damage, the presence of DM, a history of trabeculectomy, and uveitis were excluded from this multifactorial analysis. We excluded DM from the baseline factors, because DM is one of the factors underlying endothelial cell loss^52^. The following DM conditions varied among the subjects; well-controlled or poorly-controlled, insulin dependent or insulin independent, with or without ocular pathology. The remaining 102 eyes were enrolled as a subgroup of uncomplicated DSAEK cases; we conducted the same statistical analysis to evaluate the influence of variables on graft failure using Cox proportional hazard regression (univariate and multifactorial analysis).

To assess the association between postoperative ECD and all the variables, univariate analysis was conducted using Spearman’s rank correlations for each variable at 1, 3, 6, 12 and 24 months postoperatively. Multiple linear regression was also conducted using backward stepwise analysis. A decay model was evaluated that fitted the two exponentials to each set of postoperative ECDs in the groups with iris damage scores of 0, 1–2 and 3–4 as reported previously^16^. ECD = p∙exp(-at) + q∙exp(-bt), where t is time, p and q are constants whose sum equals to the preoperative graft ECD, and a and b are exponential rate constants.

To test the influence of iris damage score for different indications, we stratified the groups based on the indications for DSAEK and compared the graft survival rate among the different iris damage scores. The Kaplan Meier method was used to analyze the graft survival rate, and the log rank (Mantel Cox) test was used to evaluate P values. A P value < 0.05 was considered statistically significant. Data were expressed as means ± standard deviation (SD).

## Additional Information

**How to cite this article**: Ishii, N. *et al.* Factors associated with graft survival and endothelial cell density after Descemet's stripping automated endothelial keratoplasty. *Sci. Rep.* **6**, 25276; doi: 10.1038/srep25276 (2016).

## Supplementary Material

Supplementary Information

## Figures and Tables

**Table 1:** Demographics of all patients.

Recipient characteristics		Donor characteristics		Intra- and postoperative characteristics	
Patients, n	225	Mean age (years old ± SD)	65.2 ± 11.1	Simultaneous CS, n (%)	82 (36)
Male, n (%)	66 (29)	65≤, n (%)	129 (57)	Lens status/IOL fixation	
Mean age (years old ± SD)	69.7 ± 12.1	Imported graft, n (%)	172 (76)	Phakia	0(0)
65≤, n (%)	173 (77)	Graft ECD(cells/mm^2^)		AC IOL	0(0)
History of diabetes, n (%)	21 (9)	mean ± SD	2651 ± 322.8	Capsular bag	208(92)
Indication, n (%)		2500≤, n (%)	149 (66)	TS-IOL	17(8)
LI-BK	81 (36)	Graft diameter (mm), n (%)		Experienced surgeon, n (%)	110 (49)
PBK/ABK	54 (24)	7	4 (2)	Intraoperative iris damage	27 (12)
FECD	26 (12)	7.25	1 (1)	Postoperative re-bubbling, n (%)	
Others	64 (28)	7.5	13 (6)	None	194 (86)
Pre-existing Iris damage score, n (%)		7.75	46 (20)	Once	23 (10)
0	105 (47)	8	136 (60)	Twice	6 (3)
1	61 (27)	8.25	18 (8)	Thrice	2 (1)
2	28 (13)	8.5	7 (3)	Rejection episodes, n (%)	8 (4)
3	20 (9)				
4	11 (5)				
Previous intraocular surgeries, n(%)					
None	78 (35)				
Once	108 (48)				
Twice	30 (13)				
Thrice ＜	9 (4)				

SD: standard deviation, LI-BK: laser-iridotomy-related bullous keratopathy, PBK: pseudophakic bullous keratopathy, ABK: aphakic bullous keratopathy, FECD: Fuchs’ endothelial corneal dystrophy, ECD: endothelial cell density, CS: cataract surgery, IOL: intraocular lens, AC IOL: anterior chamber IOL, TS-IOL: transscleral IOL fixation.

**Table 2:** Association between baseline factors and graft failure in all patients.

Baseline Factors	No.	Univariate Models			Multifactorial Model		
		HR	95% CI	P Value	HR	95% CI	P Value
Gender							
Female	159	1			1		
Male	66	0.8	0.35–1.63	0.55	0.49	0.19–1.16	0.11
Age at surgery (years old)							
<65	52	1			1		
65≤	173	0.91	0.44–2.06	0.82	0.87	0.37–2.21	0.77
History of diabetes							
No	204	1			1		
Yes	21	1.13	0.27–3.15	0.85	1.08	0.22–5.14	0.92
Indication							
FECD	26	1			1		
LI-BK	81	1.24	0.39–5.43	0.73	0.99	0.27–4.79	0.99
PBK/ABK	54	0.66	0.15–3.35	0.59	0.29	0.05–1.81	0.18
Others	64	2.39	0.8–10.3	0.13	1.56	0.38–8.06	0.55
Pre-existing iris damage score							
0	105	1			1		
1 + 2	89	2.02	0.83–5.38	0.12	1.7	0.57–5.27	0.34
3 + 4	31	8.53	3.6–22.4	<0.0001	7.57	2.57–24.3	0.0002
Number of previous intraocular surgeries							
<Once	186	1			1		
Twice≤	39	2.66	1.25–5.31	0.026	1.16	0.43–3.04	0.77
Donor age (years old)							
<65	96	1			1		
65≤	129	1.54	0.78–3.19	0.22	1.32	0.6–3.01	0.49
Graft							
Domestic	53	1			1		
Imported	172	0.96	0.47–2.18	0.92	1.23	0.49–3.29	0.66
Graft ECD (cells/mm^2^)							
2500≤	149	1			1		
<2500	76	1.97	0.99–3.83	0.05	1.68	0.78–3.62	0.18
Graft diameter (mm)							
8≤	161	1			1		
<8	64	0.98	0.46–1.96	0.96	1.13	0.5–2.43	0.75
Simultaneous CS							
Yes	82	1			1		
No	143	1.78	0.87–4.02	0.12	1.43	0.58–3.78	0.45
IOL position							
Capsular bag	208	1			1		
TS-IOL	17	2.53	0.86–6.02	0.086	3.88	1.03–12.4	0.046
Experienced surgeon							
Yes	110	1			1		
No	115	1.11	0.57–2.18	0.76	2.51	1.04–6.09	0.042
Intraoperative iris damage							
No	198	1			1		
Yes	27	1.45	0.54–3.26	0.43	1.43	0.43–4.49	0.55
Postoperative re-bubbling							
No	194	1			1		
Yes	31	2	0.85–4.21	0.11	2.71	1.06–6.32	0.037
Rejection							
No	217	1			1		
Yes	8	1.53	0.25–5.04	0.58	1.49	0.22–6.04	0.63

Cox proportional hazard regression analysis.

HR: hazard ratio, CI: confidence interval, FECD: Fuchs’ endothelial corneal dystrophy, LI-BK: laser-iridotomy-related bullous keratopathy, PBK: pseudophakic bullous keratopathy, ABK: aphakic bullous keratopathy, ECD: endothelial cell density, CS: cataract surgery, IOL: intraocular lens, TS-IOL: transscleral suturing of intraocular lens.

**Table 3:**
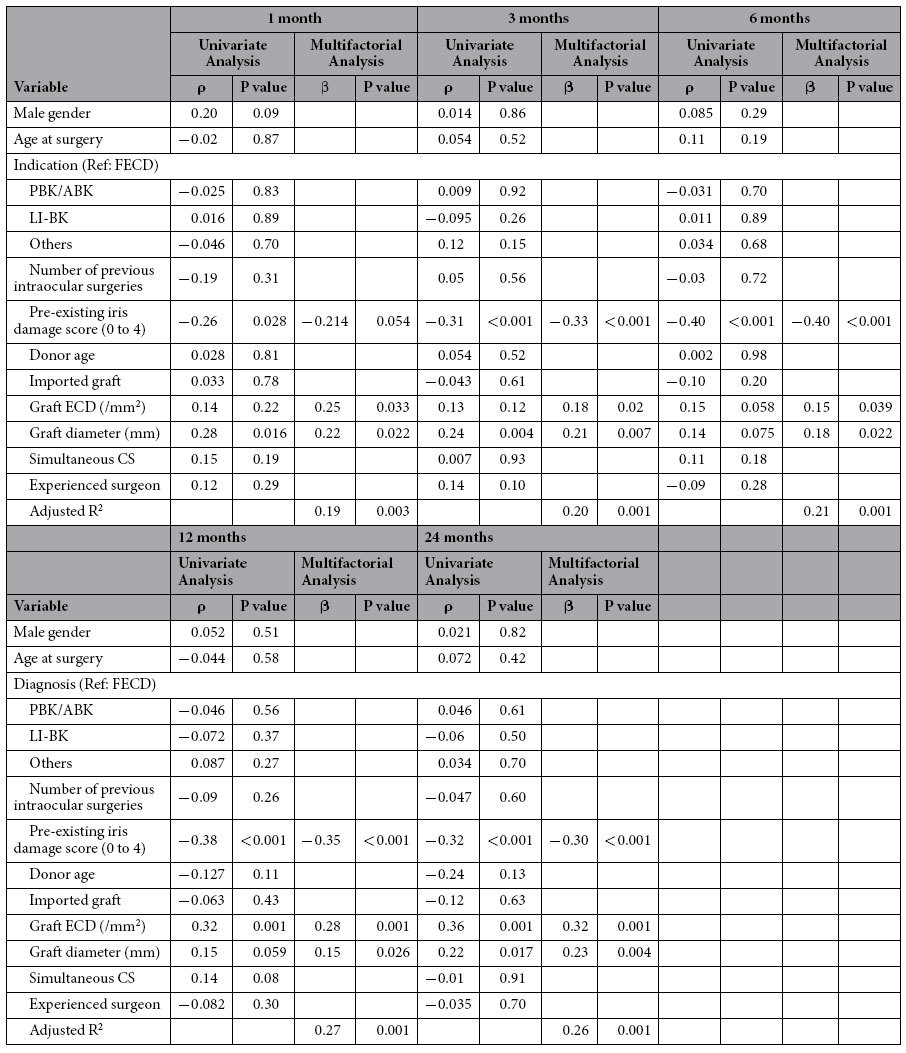
Association between baseline factors and postoperative ECD in all patients.

Linear regression analysis. FECD: Fuchs’ endothelial corneal dystrophy, PBK: pseudophakic bullous keratopathy, ABK: aphakic bullous keratopathy, LI-BK: laser-iridotomy-related bullous keratopathy, ECD: endothelial cell density, CS: cataract surgery.

## References

[b1] Price F. W. Jr. & Price M. O. Descemet’s stripping with endothelial keratoplasty in 50 eyes: a refractive neutral corneal transplant. J Refract Surg 21, 339–345 (2005). 16128330 10.3928/1081-597X-20050701-07

[b2] Price M. O. & Price F. W. Jr. Descemet’s stripping with endothelial keratoplasty: comparative outcomes with microkeratome-dissected and manually dissected donor tissue. Ophthalmology 113, 1936–1942 (2006). 16935344 10.1016/j.ophtha.2006.05.034

[b3] Koenig S. B., Covert D. J., Dupps W. J. Jr. & Meisler D. M. Visual acuity, refractive error, and endothelial cell density six months after Descemet stripping and automated endothelial keratoplasty (DSAEK). Cornea 26, 670–674 (2007). 17592314 10.1097/ICO.0b013e3180544902

[b4] Gorovoy M. S. Descemet-stripping automated endothelial keratoplasty. Cornea 25, 886–889 (2006). 17102661 10.1097/01.ico.0000214224.90743.01

[b5] Ghaznawi N. & Chen E. S. Descemet’s stripping automated endothelial keratoplasty: innovations in surgical technique. Current Opin Ophthalmol 21, 283–287 (2010). 10.1097/ICU.0b013e32833a8cc9 20467318

[b6] Patel S. V., Baratz K. H., Hodge D. O., Maguire L. J. & McLaren J. W. The effect of corneal light scatter on vision after descemet stripping with endothelial keratoplasty. Arch Ophthalmol 127, 153–160 (2009). 19204232 10.1001/archophthalmol.2008.581

[b7] Yamaguchi T. *et al.* Effect of anterior and posterior corneal surface irregularity on vision after Descemet-stripping endothelial keratoplasty. J Cataract Refract Surg 35, 688–694 (2009). 19304090 10.1016/j.jcrs.2008.11.062

[b8] Yamaguchi T. *et al.* Comparison of anterior and posterior corneal surface irregularity in Descemet stripping automated endothelial keratoplasty and penetrating keratoplasty. Cornea 29, 1086–1090 (2010). 20567202 10.1097/ICO.0b013e3181d0092c

[b9] Uchino Y. *et al.* Comparison of corneal thickness and haze in DSAEK and penetrating keratoplasty. Cornea 30, 287–290 (2011). 21045661 10.1097/ICO.0b013e3181eeafd6

[b10] Price M. O. *et al.* Descemet’s stripping automated endothelial keratoplasty: three-year graft and endothelial cell survival compared with penetrating keratoplasty. Ophthalmology 120, 246–251 (2013). 23107581 10.1016/j.ophtha.2012.08.007 PMC3562557

[b11] Ang M. *et al.* Endothelial cell loss and graft survival after Descemet’s stripping automated endothelial keratoplasty and penetrating keratoplasty. Ophthalmology 119, 2239–2244 (2012). 22885122 10.1016/j.ophtha.2012.06.012

[b12] Li J. Y., Terry M. A., Goshe J., Shamie N. & Davis-Boozer D. Graft rejection after Descemet’s stripping automated endothelial keratoplasty: graft survival and endothelial cell loss. Ophthalmology 119, 90–94 (2012). 22115709 10.1016/j.ophtha.2011.07.007

[b13] Hjortdal J., Pedersen I. B., Bak-Nielsen S. & Ivarsen A. Graft rejection and graft failure after penetrating keratoplasty or posterior lamellar keratoplasty for fuchs endothelial dystrophy. Cornea 32, e60–63 (2013). 23086366 10.1097/ICO.0b013e3182687ff3

[b14] Ezon I., Shih C. Y., Rosen L. M., Suthar T. & Udell I. J. Immunologic graft rejection in descemet’s stripping endothelial keratoplasty and penetrating keratoplasty for endothelial disease. Ophthalmology 120, 1360–1365 (2013). 23531352 10.1016/j.ophtha.2012.12.036

[b15] Lass J. H. *et al.* Baseline factors related to endothelial cell loss following penetrating keratoplasty. Arch Ophthalmol 129, 1149–1154 (2011). 21555600 10.1001/archophthalmol.2011.102 PMC4186996

[b16] Armitage W. J., Dick A. D. & Bourne W. M. Predicting endothelial cell loss and long-term corneal graft survival. Invest Ophthalmol Vis Sci 44, 3326–3331 (2003). 12882777 10.1167/iovs.02-1255

[b17] Cornea Donor Study Investigator, G. *et al.* Donor age and corneal endothelial cell loss 5 years after successful corneal transplantation. Specular microscopy ancillary study results. Ophthalmology 115, 627–632 (2008). 18387408 10.1016/j.ophtha.2008.01.004 PMC2959119

[b18] Bertelmann E., Pleyer U. & Rieck P. Risk factors for endothelial cell loss post-keratoplasty. Acta Ophthalmol 84, 766–770 (2006). 10.1111/j.1600-0420.2006.00726.x 17083535

[b19] Writing Committee for the Cornea Donor Study Research, G. *et al.* Donor age and factors related to endothelial cell loss 10 years after penetrating keratoplasty: Specular Microscopy Ancillary Study. Ophthalmology 120, 2428–2435 (2013). 24246826 10.1016/j.ophtha.2013.08.044 PMC3835371

[b20] Nishimura J. K., Hodge D. O. & Bourne W. M. Initial endothelial cell density and chronic endothelial cell loss rate in corneal transplants with late endothelial failure. Ophthalmology 106, 1962–1965 (1999). 10519593 10.1016/S0161-6420(99)90409-8

[b21] Lee H. S. & Kim M. S. Influential factors on the survival of endothelial cells after penetrating keratoplasty. Euro J Ophthalmol 19, 930–935 (2009). 10.1177/112067210901900606 19882586

[b22] Bahar I., Kaiserman I., Sansanayudh W., Levinger E. & Rootman D. S. Busin Guide vs Forceps for the Insertion of the Donor Lenticule in Descemet Stripping Automated Endothelial Keratoplasty. Am J Ophthalmol 147, 220–226 (2009). 18930446 10.1016/j.ajo.2008.08.029

[b23] Koenig S. B. & Covert D. J. Early results of small-incision Descemet’s stripping and automated endothelial keratoplasty. Ophthalmology 114, 221–226 (2007). 17156845 10.1016/j.ophtha.2006.07.056

[b24] Ang M. *et al.* Comparison of a donor insertion device to sheets glide in Descemet stripping endothelial keratoplasty: 3-year outcomes. Am J Ophthalmol 157, 1163–1169 (2014). 24582993 10.1016/j.ajo.2014.02.049

[b25] Bourne W. M., Nelson L. R. & Hodge D. O. Central corneal endothelial cell changes over a ten-year period. Invest Ophthalmol Vis Sci 38, 779–782 (1997). 9071233

[b26] Bourne W. M., Nelson L. R. & Hodge D. O. Continued endothelial cell loss ten years after lens implantation. Ophthalmology 101, 1014–1022 (1994). 8008341 10.1016/s0161-6420(94)31224-3

[b27] Ing J. J., Ing H. H., Nelson L. R., Hodge D. O. & Bourne W. M. Ten-year postoperative results of penetrating keratoplasty. Ophthalmology 105, 1855–1865 (1998). 9787355 10.1016/S0161-6420(98)91030-2

[b28] Bourne W. M., Hodge D. O. & Nelson L. R. Corneal endothelium five years after transplantation. Am J Ophthalmol 118, 185–196 (1994). 8053464 10.1016/s0002-9394(14)72898-3

[b29] Zacks C. M., Abbott R. L. & Fine M. Long-term changes in corneal endothelium after keratoplasty. A follow-up study. Cornea 9, 92–97 (1990). 2328589

[b30] Rapuano C. J. *et al.* Results of alloplastic tube shunt procedures before, during, or after penetrating keratoplasty. Cornea 14, 26–32 (1995). 7712732

[b31] Hollander D. A. *et al.* Graft failure after penetrating keratoplasty in eyes with Ahmed valves. Am J Ophthalmol 150, 169–178 (2010). 20537311 10.1016/j.ajo.2010.02.014

[b32] Numa A., Nakamura J., Takashima M. & Kani K. Long-term corneal endothelial changes after intraocular lens implantation. Anterior vs posterior chamber lenses. Jap J Ophthalmol 37, 78–87 (1993). 8320869

[b33] Bromley J. G., Randleman J. B., Stone D., Stulting R. D. & Grossniklaus H. E. Clinicopathologic findings in iridocorneal endothelial syndrome and posterior polymorphous membranous dystrophy after Descemet stripping automated endothelial keratoplasty. Cornea 31, 1060–1064 (2012). 22333668 10.1097/ICO.0b013e31823fb978

[b34] Pezzi P. P., Marenco M., Cosimi P., Mannino G. & Iannetti L. Progression of essential iris atrophy studied with confocal microscopy and ultrasound biomicroscopy: a 5-year case report. Cornea 28, 99–102 (2009). 19092417 10.1097/ICO.0b013e3181822579

[b35] Ambrose V. M., Walters R. F., Batterbury M., Spalton D. J. & McGill J. I. Long-term endothelial cell loss and breakdown of the blood-aqueous barrier in cataract surgery. J Cataract Refract Surg 17, 622–627 (1991). 1941598 10.1016/s0886-3350(13)81052-8

[b36] Takase H. *et al.* Comparison of the ocular characteristics of anterior uveitis caused by herpes simplex virus, varicella-zoster virus, and cytomegalovirus. Jap J Ophthalmol 58, 473–482 (2014). 25124341 10.1007/s10384-014-0340-6

[b37] Grzetic-Lenac R., Merlak M., Balog T., Markusic V. & Dekaris I. The expression of interleukin-1 alpha, TNF and VEGF in corneal cells of patients with bullous keratopathy. Collegium antropologicum 35 Suppl 2, 171–173 (2011). 22220428

[b38] Inoue T., Kawaji T. & Tanihara H. Monocyte chemotactic protein-1 level in the aqueous humour as a prognostic factor for the outcome of trabeculectomy. Clin Experiment Ophthalmol 42, 334–341 (2014). 24025148 10.1111/ceo.12204

[b39] Holland S. P., Morck D. W. & Lee T. L. Update on toxic anterior segment syndrome. Current Opin in Ophthalmol 18, 4–8 (2007). 10.1097/ICU.0b013e3280117d0c 17159439

[b40] Mamalis N. Toxic anterior segment syndrome. J Cataract Refract Surg 32, 181–182 (2006). 16564962 10.1016/j.jcrs.2006.01.036

[b41] Sharma A. *et al.* Persistent corneal edema after collagen cross-linking for keratoconus. Am J Ophthalmol 154, 922–926 (2012). 22959362 10.1016/j.ajo.2012.06.005

[b42] Yazu H., Yamaguchi T., Ishii N., *et al.* Descemet’s Stripping Automated Endothelial Keratoplasty in Eyes with Transscleral Suture of Intraocular Lens. J Cataract Refract Surg 42 (2016). 10.1016/j.jcrs.2016.02.044 27373391

[b43] Hirayama M., Yamaguchi T., Satake Y. & Shimazaki J. Surgical outcome of Descemet’s stripping automated endothelial keratoplasty for bullous keratopathy secondary to argon laser iridotomy. Graefes Arch Clin Exp Ophthalmol. 250, 1043–1050 (2012). 10.1007/s00417-012-1927-6 PMC3396288 22286710

[b44] Romano V. *et al.* Influence of graft size on graft survival following Descemet stripping automated endothelial keratoplasty. Br J Ophthalmol 99, 784–788 (2015). 25583280 10.1136/bjophthalmol-2014-305648

[b45] Kim P., Yeung S. N., Lichtinger A., Amiran M. D. & Rootman D. S. Descemet stripping automated endothelial keratoplasty using infant donor tissue. Cornea 31, 52–54 (2012). 22157523 10.1097/ICO.0b013e31821eeac0

[b46] Kobayashi A., Yokogawa H., Yamazaki N., Masaki T. & Sugiyama K. Endothelial keratoplasty with infant donor tissue. Clin Ophthalmol 8, 1827–1830 (2014). 25246761 10.2147/OPTH.S68618 PMC4166343

[b47] Terry M. A., Li J., Goshe J. & Davis-Boozer D. Endothelial keratoplasty: the relationship between donor tissue size and donor endothelial survival. Ophthalmology 118, 1944–1949 (2011). 21652077 10.1016/j.ophtha.2011.02.023

[b48] Heinzelmann S., Böhringer D., Eberwein P., Reinhard T. & Maier P. Outcomes of Descemet membrane endothelial keratoplasty, Descemet stripping automated endothelial keratoplasty and penetrating keratoplasty from a single centre study. Graefes Arch Clin Exp Ophthalmol. 254, 515–522 (2016). 26743748 10.1007/s00417-015-3248-z

[b49] Nahum Y., Mimouni M. & Busin M. Risk Factors Predicting the Need for Graft Exchange After Descemet Stripping Automated Endothelial Keratoplasty. Cornea 34, 876–879 (2015). 26020823 10.1097/ICO.0000000000000455

[b50] Anshu A., Price M. O. & Price F. W. Jr. Descemet’s stripping endothelial keratoplasty under failed penetrating keratoplasty: visual rehabilitation and graft survival rate. Ophthalmology 118, 2155–2160 (2011). 21906816 10.1016/j.ophtha.2011.04.032

[b51] Kobayashi A. *et al.* Descemet stripping with automated endothelial keratoplasty for bullous keratopathies secondary to argon laser iridotomy–preliminary results and usefulness of double-glide donor insertion technique. Cornea 27, Suppl 1, S62–69 (2008). 18813077 10.1097/ICO.0b013e31817f38e9

[b52] Yamazoe K. *et al.* Outcomes of cataract surgery in eyes with a low corneal endothelial cell density. J Cataract Refract Surg 37, 2130–2136 (2011). 21908173 10.1016/j.jcrs.2011.05.039

