# Supplementary material for: Factors associated with graft survival and endothelial cell density after Descemet’s stripping automated endothelial keratoplasty
Source: Sci Rep. 2016 Apr 28;6:25276. doi: 10.1038/srep25276 (PMC4848492; doi:10.1038/srep25276)
Supplement: Supplementary Information [file srep25276-s1.doc]

**Factors associated with graft survival and endothelial cell density**

**after Descemet’s stripping automated endothelial keratoplasty**

Nobuhito Ishii, Takefumi Yamaguchi, Hiroyuki Yazu,

Yoshiyuki Satake, Akitoshi Yoshida, Jun Shimazaki

Supplementary Table S1. Demographics of 102 uncomplicated cases

| Recipient characteristics | | Donor characteristics | | Intra- and postoperative characteristics | |
| --- | --- | --- | --- | --- | --- |
| Patients, n | 102 | Mean age (years old ± SD) | 65.1 ± 12 | Simultaneous CS, n (%) | 38 (37) |
| Male, n (%) | 24 (24) | 65≤, n (%) | 56 (55) | By experienced surgeon, n (%) | 47 (46) |
| Mean age (year old ± SD) | 71.6 ± 10.1 | Imported graft, n (%) | 76 (74) | Intraoperative iris damage | 0 (0) |
| 65≤, n (%) | 82 (80) | Graft ECD (cells/mm^2^) |  |  |  |
| DM, n (%) | 0 (0) | mean ± SD | 2635 ± 322 | Postoperative re-bubbling, n (%) |  |
| Indication, n (%) |  | 2500≤, n (%) | 64 (63) | None | 102 (100) |
| LI-BK | 48 (47) | Graft size (mm) | n (%) | Once | 0 (0) |
| PBK/ABK | 38 (37) | 7 | 2 (2) | Twice | 0 (0) |
| FECD | 16 (16) | 7.25 | 1 (1) | Thrice | 0 (0) |
| Others | 0 (0) | 7.5 | 7 (7) |  |  |
| Pre-existing Iris damage score, n (%) | | 7.75 | 22 (22) | Rejection episodes, n (%) | 0 (0) |
| 0 | 62 (61) | 8 | 62 (61) |  |  |
| 1 | 25 (25) | 8.25 | 5 (5) |  |  |
| 2 | 6 (6) | 8.5 | 3 (3) |  |  |
| 3 | 8 (8) |  |  |  |  |
| 4 | 1 (1) |  |  |  |  |

SD: standard deviation, FECD: Fuchs’ endothelial corneal dystrophy, LI-BK: laser-iridotomy-related bullous keratopathy, PBK: pseudophakic bullous keratopathy, ABK: aphakic bullous keratopathy, ECD: endothelial cell density, CS: cataract surgery

Supplementary Table S2. Association between baseline factors and graft failure in 102 uncomplicated cases

| Baseline Factors | Univariate Models | | | | Multifactorial Models | | |
| --- | --- | --- | --- | --- | --- | --- | --- |
|  | No. | HR | 95% CI | P Value | HR | 95% CI | P Value |
| Gender |  |  |  |  |  |  |  |
| Female | 78 | 1 |  |  | 1 |  |  |
| Male | 24 | 1.15 | 0.17-4.98 | 0.87 | 2.39 | 0.41-18.2 | 0.40 |
| Age at surgery (years old) |  |  |  |  |  |  |  |
| <65 | 20 | 1 |  |  | 1 |  |  |
| 65≤ | 82 | 0.4 | 0.09-1.94 | 0.23 | 0.33 | 0.06-2.43 | 0.31 |
| Indication |  |  |  |  |  |  |  |
| FECD | 16 | 1 |  |  | 1 |  |  |
| LI-BK | 48 | 0.58 | 0.11-4.18 | 0.54 | 1.26 | 0.13-11.9 | 0.84 |
| PBK/ABK | 38 | 0.37 | 0.05-8.89 | 0.54 | 0.39 | 0.004-4.9 | 0.40 |
| Pre-existing iris damage score |  |  |  |  |  |  |  |
| 0 | 62 | 1 |  |  | 1 |  |  |
| 1-2 | 31 | 0.44 | 0.02-2.99 | 0.43 | 1.08 | 0.13-9,23 | 0.94 |
| 3-4 | 9 | 5.54 | 1.08-25.4 | 0.04 | 13.6 | 1.54-119 | 0.019 |
| Donor age (years old) |  |  |  |  |  |  |  |
| <65 | 46 | 1 |  |  | 1 |  |  |
| 65≤ | 56 | 0.47 | 0.09-1.93 | 0.29 | 0.13 | 0.26-1.76 | 0.57 |
| Graft |  |  |  |  |  |  |  |
| Domestic | 26 | 1 |  |  | 1 |  |  |
| Imported | 76 | 0.37 | 0.08-1.6 | 0.18 | 0.33 | 0.07-1.64 | 0.18 |
| Graft ECD (cells/mm^2^) |  |  |  |  |  |  |  |
| 2500≤ | 64 | 1 |  |  | 1 |  |  |
| <2500 | 38 | 1.97 | 0.47-8.36 | 0.34 | 2.73 | 0.09-1.95 | 0.27 |
| Graft size (mm) |  |  |  |  |  |  |  |
| 8≤ | 70 | 1 |  |  | 1 |  |  |
| <8 | 32 | 3.36 | 0.82-16.4 | 0.09 | 4.81 | 0.84-28.4 | 0.08 |
| Simultaneous CS |  |  |  |  |  |  |  |
| Yes | 38 | 1 |  |  | 1 |  |  |
| No | 64 | 3.97 | 0.7-74.3 | 0.13 | 3.11 | 0.43-22.2 | 0.26 |
| Experienced surgeon |  |  |  |  |  |  |  |
| Yes | 47 | 1 |  |  | 1 |  |  |
| No | 55 | 1.43 | 0.35-7.01 | 0.61 | 1.44 | 0.28-7.24 | 0.18 |

Cox proportional hazard regression analysis was made.

HR: hazard ratio, CI: confidence interval, FECD: Fuchs’ endothelial corneal dystrophy, LI-BK: laser-iridotomy-related bullous keratopathy, PBK: pseudophakic bullous keratopathy, ABK: aphakic bullous keratopathy, ECD: endothelial cell density, CS: cataract surgery

|  | 1 month | | | | | 3 months | | | | 6 months | | | |
| --- | --- | --- | --- | --- | --- | --- | --- | --- | --- | --- | --- | --- | --- |
|  | Univariate Analysis | | Multifactorial Analysis | | Univariate Analysis | | | Multifactorial Analysis | | Univariate Analysis | | Multifactorial Analysis | |
| Variable | ρ | P value | Β | P value | ρ | | P value | β | P value | ρ | P value | β | P value |
| Male gender | 0.14 | 0.39 |  |  | -0.11 | | 0.37 |  |  | 0.074 | 0.55 |  |  |
| Age at surgery | 0.14 | 0.39 |  |  | 0.29 | | 0.014 | 0.244 | 0.024 | 0.28 | 0.022 |  |  |
| Indication (Ref: FECD) |  |  |  |  |  | |  |  |  |  |  |  |  |
| PBK/ABK | -0.0024 | 0.99 |  |  | 0.011 | | 0.93 |  |  | 0.048 | 0.70 |  |  |
| LI-BK | -0.054 | 0.74 |  |  | 0.0065 | | 0.96 |  |  | 0.017 | 0.89 |  |  |
| Number of previous intraocular surgery | -0.17 | 0.49 |  |  | -0.03 | | 0.79 |  |  | -0.09 | 0.48 |  |  |
| Pre-existing iris damage score (0 to 4) | -0.35 | 0.028 | -0.30 | 0.055 | -0.38 | | 0.001 | -0.40 | 0.001 | -0.32 | 0.0097 | -0.43 | 0.001 |
| Donor age | 0.09 | 0.58 |  |  | 0.10 | | 0.39 |  |  | 0.028 | 0.82 |  |  |
| Imported graft | 0.13 | 0.42 |  |  | 0.032 | | 0.79 |  |  | 0.018 | 0.89 |  |  |
| Graft ECD (/mm^2^) | 0.11 | 0.49 |  |  | 0.15 | | 0.20 |  |  | 0.20 | 0.11 | 0.24 | 0.024 |
| Graft size (mm) | 0.27 | 0.087 | 0.29 | 0.064 | 0.21 | | 0.08 |  |  | 0.30 | 0.013 | 0.36 | 0.001 |
| Simultaneous CS | 0.22 | 0.17 |  |  | 0.11 | | 0.37 |  |  | 0.15 | 0.22 |  |  |
| Experienced surgeon | 0.20 | 0.20 |  |  | 0.18 | | 0.14 |  |  | -0.018 | 0.88 |  |  |
| Adjusted R^2^ |  |  | 0.16 | 0.014 |  | |  | 0.19 | 0.001 |  |  | 0.31 | <0.0001 |
|  |  |  |  |  |  | |  |  |  |  |  |  |  |
|  | 12 months | | | | 24 months | | | | |  | | | |
|  | Univariate Analysis | | Multifactorial Analysis | | Univariate Analysis | | | Multifactorial Analysis | |  | | | |
| Variable | ρ | P value | β | P value | ρ | | P value | β | P value |  | | | |
| Male gender | -0.015 | 0.90 |  |  | -0.082 | | 0.53 |  |  |  | | | |
| Age at surgery | 0.18 | 0.12 |  |  | 0.27 | | 0.034 | 0.26 | 0.036 |  | | | |
| Diagnosis (Ref: FECD) |  |  |  |  |  | |  |  |  |  | | | |
| PBK/ABK | 0.034 | 0.78 |  |  | 0.11 | | 0.42 |  |  |  | | | |
| LI-BK | -0.059 | 0.62 |  |  | 0.067 | | 0.61 |  |  |  | | | |
| Number of previous intraocular surgery | -0.20 | 0.084 |  |  | -0.16 | | 0.24 |  |  |  | | | |
| Pre-existing iris damage score (0 to 4) | -0.29 | 0.012 | -0.36 | 0.001 | -0.13 | | 0.32 |  |  |  | | | |
| Donor age | -0.015 | 0.90 |  |  | -0.011 | | 0.93 |  |  |  | | | |
| Imported graft | -0.083 | 0.49 |  |  | -0.17 | | 0.19 |  |  |  | | | |
| Graft ECD (/mm^2^) | 0.28 | 0.018 | 0.28 | 0.01 | 0.25 | | 0.051 | 0.26 | 0.037 |  | | | |
| Graft size (mm) | 0.19 | 0.11 | 0.26 | 0.014 | 0.22 | | 0.091 |  |  |  | | | |
| Simultaneous CS | 0.14 | 0.24 |  |  | 0.002 | | 0.99 |  |  |  | | | |
| Experienced surgeon | -0.074 | 0.54 |  |  | 0.041 | | 0.76 |  |  |  | | | |
| Adjusted R^2^ |  |  | 0.24 | 0.001 |  | |  | 0.11 | 0.015 |  | | | |

Supplementary Table S3. Association between baseline factors and postoperative ECD in 102 uncomplicated cases.

Linear regression analysis.

FECD: Fuchs’ endothelial corneal dystrophy, PBK: pseudophakic bullous keratopathy, ABK: aphakic bullous keratopathy, LI-BK: laser-iridotomy-related bullous keratopathy, ECD: endothelial cell density, CS: cataract surgery
